# Observation and Control of Single-Component Adhesion Interphase of Polyamide 66 through Confocal Raman Microspectroscopy

**DOI:** 10.1021/acsami.4c18513

**Published:** 2024-12-26

**Authors:** Takuya Matsumoto, Naoki Shimoura, Naho Aoki, Naoto Takahashi, Shun Mizuno, Takashi Nishino

**Affiliations:** Department of Chemical Science and Engineering, Graduate School of Engineering, Kobe University, Rokko, Nada Kobe 657-8501, Japan

**Keywords:** adhesion, polyamide 66, interphase, confocal Raman spectroscopy, crystallization, deuterated polymer

## Abstract

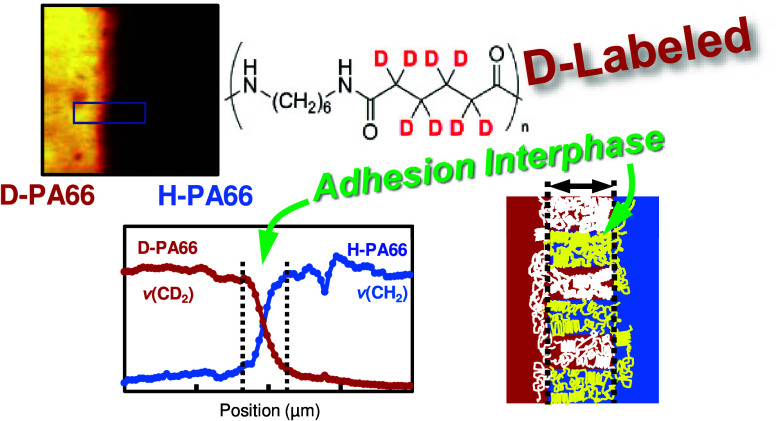

Manufacturing using adhesion technology has attracted much attention. Examples of adhesion include the lay-up of carbon fiber reinforced thermoplastic prepregs and the lamination of food packaging. In single-component adhesion systems, the analysis of the boundary region poses challenges because of the absence of chemical and physical discrimination at the adhesion interphase. Polyamide 66, one of the typical engineering plastics, is widely accepted as a structural material in automobiles and packaging films. Therefore, finer control of adhesion with polyamide 66 is crucial for advancing adhesion manufacturing. In this work, we focused and investigated the interphase of a single-component adhesion system with polyamide 66. For the analyses of single-component polyamide 66 laminates, an adhesion system with nondeuterated and deuterated polyamides was utilized, and their interphase structures were evaluated by confocal Raman microspectroscopy. The interphase region of the adhesion specimens was able to be characterized and evaluated, revealing an expansion to a thickness of several micrometers. The interphase thickness was increased with thermal annealing postlamination, whereas no thickness increase was observed in adhered specimens using the polyamide 66 substrates through thermal crystallization before lamination. The formation of the interphase region can be attributed to the crystal growing and lamella interlocking in the boundary region. Moreover, the larger interphase thickness was strongly associated with an increase in adhesion fracture toughness. These results suggested that the adhesion properties of crystalline substrates were decided by crystallization behavior and the thermal annealing process, even when using the same component adhesion systems.

## Introduction

Adhesion technologies have been developed in the fields of automobiles and aircrafts, as well as household items and food packaging.^1−5^ In the products using the adhesion system, interfaces are generated at the boundary region between the adherent materials. Various interactions and phenomena are involved at the interface between polymer materials: mechanical anchoring and zipping,^6−10^ diffusion and entanglement of polymer chains,^11−18^ hydrogen bonding,^19−21^ electrostatic interactions,^22−25^ ionic interactions,^26−28^ van der Waals forces,^29−31^ covalent bonding,^32−40^ etc. It has been reported that for proper and dependable adhesion systems, their various interactions were controlled, and the adhesion strengths were increased through multiple approaches. In the investigation and clarification of the adhesion mechanism for the control of the adhesion properties, analyses of the structure and physical properties at the interface are indispensable. Deeper insights into the interface directly improve the reliability of the adhesion.

Melt-lamination is one of the typical adhesion techniques where substrates are integrated through a melting process at only their surface region without any adhesives. This lamination process involves integrating substrates with different polymer components as well as those composed of the same polymers. Even in the lamination with the same polymer substrates, an adhesion interface with single polymer components is formed. The single-component interfaces have been observed in the case of packaging for foods or snacks.^41,42^ Moreover, in the manufacturing process of polymer materials, weld lines also possessed interfaces with the single components.^43^ In the case of the lay-up of carbon fiber reinforced thermoplastic (CFRTP) prepregs^44^ and additive manufacturing,^45−47^ the single-component interface of the layered structure significantly influences the mechanical properties of the final products. Because the properties of interfaces decide the product performance, their controls are desired. In the evaluation of the interface with different components, the interface was clearly identified through various measurement methods: X-ray diffraction,^48,49^ Fourier transform infrared (FT-IR) spectroscopy,^27,50−52^ Raman spectroscopy,^16,18,53−55^ X-ray photoelectron spectroscopy (XPS),^56,57^ sum-frequency generation spectroscopy,^25,34−36,58−60^ and so on. However, the single-component interface poses challenges because the polymer at the interface possesses the same chemical structure, microstructure, and physical properties. Therefore, to address this issue, the usage of deuterated polymers has been suggested for the analyses of the single-component interface.^12,18,61,62^ The deuterated polymers possess the chemical structure in which the hydrogen atoms of nondeuterated polymers are replaced with deuterium atoms. While these polymers exhibit nearly identical physical properties to their nondeuterated counterparts, spectroscopic identification is facilitated by the fact that deuterium atoms have twice the weight of hydrogen, and the vibration mode of the functional group with deuterium atoms is drastically changed.^18,63^ The analyses of the adhesion interface with single components are available using this deuterated polymer method.

Recently, we have suggested and proved that the adhesion interface of crystalline polymer *isotactic* polypropylene substrates expanded spatially to a three-dimensional region, rather than a two-dimensional one, with a considerable thickness.^54,55^ Therefore, the region was regarded as an “Interphase”^54,64−74^ in the adhesion systems using adhesives in the broad sense. We suspected that, at the interphase, the growth of lamella of the polymer substrates would form the interphase and have large impacts on their adhesion properties. However, in our previous research,^18,54,55^ we focused solely on the crystallization of the polymer substrates because the adhesives were amorphous or slightly crystalline. Some researchers have highlighted polymer chain diffusion at the interphase, specifically in amorphous states.^75,76^ Therefore, the crystallization behaviors of both adherent substrates at the adhesion interphase remain largely unexplored. The analysis also delved into the crystalline structure and crystallization behavior within the single-component interphase of crystalline polymers in a miscible polymer system.

Herein, we focused on the interphase of laminated polyamide 66 (PA66). We performed the analyses of the laminated interphase of polyamide 66 using deuterated polyamide 66 (D-PA66) and investigated the effect of the crystallization behaviors on the interphase structure. PA66 is one of the typical engineering plastics with high melting temperature exceeding 200 °C and served as a polymer matrix in CFRTP.^77,78^ Therefore, the single-component interphase with polyamide 66 has garnered much attention in the industrial field, and its analysis is going to develop the reliability for the adhesion system.

## Experimental Section

### Materials

All of the chemicals were purchased from chemical companies. In preparation of substrates for wedge tests, polyamide 66 pellets (Sigma-Aldrich Co., intrinsic viscosity [η]: 1.56 dL/g, viscosity average molecular weight *M*_v_: 61.5k) were employed. Deionized water was prepared by using an Elix water purification system (Merck Millipore Co., Ltd., Elix Essential 3). ^1^H NMR (400 MHz) measurements were performed with a JEOL ECZ400 (JEOL Co. Ltd.), and samples were dissolved into 2,2,2-trifluoroethanol-d3 (Sigma-Aldrich Co.).

### Synthesis of Nondeuterated PA66 (H-PA66) and Deuterated PA66 (D-PA66)

H-PA66 was synthesized by the interface polycondensation methods. First, 9.6 mL of 1,6-hexamethylene diamine chloride and 12.8 g of sodium carbonate were dissolved into 320 mL of water, and the aqueous solution of hexamethylene diamine chloride was obtained. Into 640 mL of hexane was added 9.6 mL of adipoyl chloride, and the hexane solution was prepared. The hexane solution was slowly poured onto the aqueous solution, maintaining the interface between aqueous and organic layers. H-PA66 was synthesized at the interface. H-PA66 was picked and rolled up. The obtained H-PA66 was rinsed with methanol and water. After that, H-PA66 was dissolved into phenol and poured into methanol for reprecipitation. After two reprecipitations, the obtained sample (H-PA66) powder was dried at 40 °C for 48 h under vacuum. The specific viscosity of the obtained H-PA66 was evaluated in the *o*-chlorophenol solution.^79^ The intrinsic viscosity [η] of the obtained H-PA66 was 1.35 dL/g. The viscosity average molecular weight *M*_v_ was 48.4k.^80^

In the case of deuterated polyamide 66, we used deuterated adipoyl-*d8* chloride and synthesized the polymer (D-PA66) by the same method. The intrinsic viscosity [η] of D-PA66 was 1.35 dL/g. The viscosity average molecular weight *M*_v_ was 48.4k.

Both synthesized polymers H-PA66 and D-PA66 were characterized by ^1^H NMR measurements. Their data are shown below.

H-PA66 ^1^H NMR (400 MHz, δ/ppm): 3.21–3.15 (br, 4.0H); 2.23–2.17 (br, 4.0H); 1.62–1.55 (br, 4.0H); 1.55–1.46 (br, 4.0H); 1.36–1.30 (br, 4.0H).

D-PA66 ^1^H NMR (400 MHz, δ/ppm): 3.21–3.15 (br, 4.0H); 1.55–1.46 (br, 4.0H); 1.36–1.30 (br, 4.0H).

### Preparation of Laminated PA66 Specimens for Raman Spectroscopic Measurements

0.20 g of the obtained polymer powder was dissolved into 10 mL of 1,1,1,3,3,3-hexafluoro-2-propanol (HFIP), and a 2.0 wt % solution was prepared. The solution was poured into a Teflon container, and the solvent was removed after drying at room temperature for 24 h. After further drying for 24 h at 40 °C, the substrates were trimmed with 20 × 20 mm of width and length and were obtained as s-H-PA66 and s-D-PA66. The thickness of the substrate was approximately 200 μm. The obtained substrates were annealed at 150 °C for 8 h in a convection oven, and the samples were c-H-PA66 and c-D-PA66.

The prepared substrates of s(or c)-H-PA66 and s(or c)-D-PA66 were laminated at 250 °C, 6 MPa for 30 s, using a hot-pressing machine (Gonno Hydraulic Machinery Mfg. Co., Ltd.). After lamination, the laminated films were cooled rapidly using ice water. The laminated specimen was obtained as s/q(or c/q)-Lm. The thermal annealing process was performed at 150 °C for 8 h under a vacuum. The laminated specimen was obtained as s/a(or c/a)-Lm.

**Figure 1:**
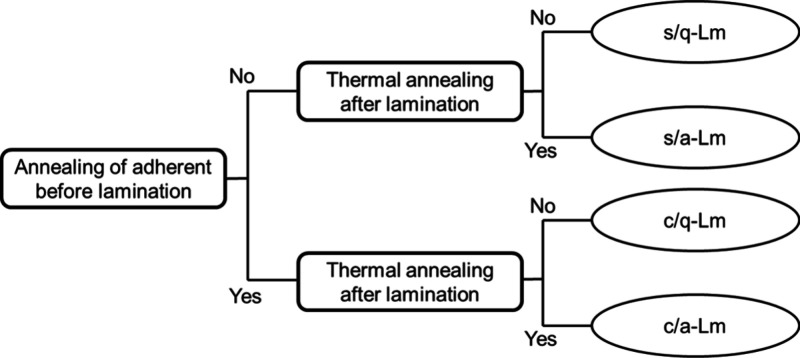
Fabrication conditions of the prepared PA66 laminates.

All the laminates and the fabrication conditions in this study were summarized in Table 1 and Figure 1. The lamination temperature and time were optimized to avoid thermal deformation of the substrates. The thermal annealing process was performed at a higher temperature than the glass transition one and lower than the melting one.

**Table 1:** Fabricated Laminated Samples for Raman Spectroscopy and Adhesion Tests

Sample	Annealing of adherent before lamination	Thermal annealing after lamination
s/q-Lm	None (s)	None (q)
s/a-Lm	None (s)	150 °C for 8 h (a)
c/q-Lm	150 °C for 8 h (c)	None (q)
c/a-Lm	150 °C for 8 h (c)	150 °C for 8 h (a)

### Preparation of PA66/PA66 Laminate Films for Wedge Tests

PA66 substrates with a 200 μm thickness were prepared with a hot-pressing machine (Gonno Hydraulic Machinery Manufacturing Co., Ltd.) at 275 °C and 6 MPa for 5 min. After that, PA66 substrates were slowly cooled to room temperature and were obtained as s-PA66. The annealing process was performed at 150 °C for 8 h, and c-PA66 was obtained. Their X-ray diffraction (XRD) profiles are shown in Figure S1 in the Supporting Information.

All of the PA66 substrates were trimmed into 10 × 50 mm rectangles. Two PA66 films were laminated by pressing at 250 °C and 3 MPa for 30 s with a hot-pressing machine (Gonno Hydraulic Machinery Mfg. Co., Ltd.) and quenching in ice water. The laminated area was 10 mm in width and 45 mm in height. Then, s/q-Lm and c/q-Lm laminates for wedge tests were obtained. To prepare s/a-Lm and c/a-Lm samples, their laminated films were annealed at 150 °C for 8 h.

Table 1 shows the four types of PA66/PA66 laminate films prepared in this study.

### Thermogravimetry (TG) Measurements

Thermogravimetric analysis (TGA) of H-PA66 and D-PA66 was performed using a Thermo plus EVOII TG8121 thermal analyzer under a nitrogen flow. The heating rate was 10 °C/min, and the temperature varied from room temperature to 500 °C. The temperature at which 5 wt % of the sample was lost was defined as the thermal decomposition temperature (*T*_d5_).

### Differential Scanning Calorimetry (DSC) Measurements

DSC measurements were performed with a differential scanning calorimeter (Thermo plus EVOII: DSC8230, RIGAKU Co. Ltd.) under a nitrogen flow. The samples were set in Al pans, and the scanning temperature varied from room temperature to 300 °C. The heating and cooling speed were 10 °C/min. The crystallinity was calculated from eq 1
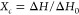
1where *ΔH* is the melting enthalpy of the sample, and Δ*H*_0_ is the equilibrium enthalpy of melting of PA66. The value of Δ*H*_0_ is 188.28 J/g.^81^

### Measurements of X-ray Diffraction

X-ray diffraction (XRD) profiles of c-H-PA66 and c-D-PA66 were measured using a Rint2000 (Rigaku Co.) in the θ/2θ method. The X-ray beam was generated at 40 kV and 20 mA, and the wavelength was 1.5418 Å (CuKα). The scanning rate was 2°/min. The stepping angle was 0.02°, and the scanning range was from 3° to 30° in 2θ.

### Measurements of Dynamic Contact Angles and Surface Free Energy

To investigate the wettability of H-PA66 and D-PA66, the dynamic contact angles of deionized water and methylene iodide were measured at room temperature using an optical microscope (HIROX Co. Ltd.). The advancing contact angle (*θ*_*a*_) was measured, while the droplet (approximately 1 mm in diameter) was enlarged (<2 mm in diameter) upon injecting the liquid from a microsyringe onto the film surface until the contact area increased. The receding contact angle (*θ*_*r*_) was measured when the contact area decreased when the liquid was slowly withdrawn.

In order to evaluate the wettability of the film surface, the average contact angle (*θ*_*av*_) was calculated by using eq 2
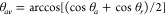
2

The surface free energy, γ_s_, was calculated from the contact angles, using eqs 3 and 4(82,83)
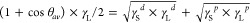
3
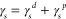
4where γ_L_ is the surface free energy of the liquid, and γ_L_^*d*^ and γ_L_^*p*^ are the dispersion and polar components of the liquid, respectively. The γ_L_^*d*^ and γ_L_^p^ values of water are 21.8 and 51.0 mJ/m^2^, respectively, and those of methylene iodide are 48.5 and 2.3 mJ/m^2^, respectively.^84^ γ_s_^*d*^ and γ_s_^*p*^ are the dispersion and polar components of the surface free energies of the substrate, respectively.

### Measurements of Wedge Tests

As shown in Figure S2 in the Supporting Information, adhesive strength of their PA66 laminates was evaluated by wedge tests in terms of the fracture toughness, *G*_*c*_. In the wedge test, a single-edged razor blade with a thickness of 78 μm was inserted at the interface of two PA66 films until the razor blade reached 10 mm from the sample edge. After 24 h, the crack opening length between the edge of the blade and the crack tip was measured using an optical microscope (HIROX Co., Ltd.) Then, the blade was inserted two times by an additional 5 mm. The opening lengths were measured 24 h, 48 h, and 72 h after wedging, and the obtained values of the opening lengths were averaged. Each sample was measured with more than 6 specimens. The fracture toughness, *G*_*c*_, was calculated by the following equation^85^
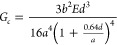
5where *E* is the Young’s modulus of PA66 film, *a* is the crack opening length between the edge of the blade and the crack tip, *b* is the thickness of the razor blade, and *d* is the average thickness of PA66 film. In this test, the symmetric structure of the laminated samples, elastic deformation of the substrates, and crack-propagation at the interphase were required. *E* was measured by using a tensile tester (AUTOGRAPH AG-X plus, Shimadzu Corporation) at the crosshead speed of 1 mm/min at room temperature. The film size was 5 mm in width and 40 mm in height. The observed Young’s modulus was averaged over at least 5 experiments for every sample.

### Fourier Transform Infrared Spectroscopic (FT-IR) Measurements

The equipment used in the FT-IR measurements was a Spectrum GX FT-IR System I-KS (PerkinElmer Co.). The samples were dispersed in KBr pellets. The wavenumber range was from 400 to 4000 cm^–1^. The resolution of wavenumber was 4 cm^–1^, and the accumulation was 10 times.

### Confocal Raman Spectroscopic Measurements

The laminated H-PA66/D-PA66 specimens were trimmed using a razor with a 10 × 5 mm rectangle. The surface of their cross section was polished mechanically with a polisher (SBT920, Meiwa Co. Ltd.) in the wet state at 100 rpm. After polishing, the specimens were washed with sonication in ethanol.

In the single spot measurements, Raman spectra of H-PA66 and D-PA66 substrates were measured in the single spot mode using a confocal Raman spectroscope (WITec K. K., Alpha 300R). A Nd/YAG semiconductor laser (wavelength: 532 nm) was used as an excitation laser. The laser intensity was 10 mW, the magnification of the objective lens with a numerical aperture of 0.75 was 50×, the exposure time was 1 s, the accumulation was 10 times, and the diffraction grating was 600 gr/mm.

2D mapping measurements were performed with the same laser, laser intensity, and diffraction grating as those in the single spot measurements. The magnification of objective lens with numerical aperture of 0.90 was 100×, the exposure time was 0.1 s, the accumulation was 1 time, the scanning area was 20 μm × 20 μm, and the stepping number was 75 spots × 75 spots. The 2D imaging was plotted based on the area intensity of CD_2_ vibration bands from 2050 to 2350 cm^–1^.^86,87^ The baselines were set based on the intensity of the ranges of 500 cm^–1^-800 cm^–1^, 1800 cm^–1^-2010 cm^–1^, and 2550 cm^–1^-2700 cm^–1^.

In the line scanning measurements, we evaluated the laminated interface across the interface of H-PA66/D-PA66 laminates with the same laser, intensity, and diffraction grating as those in the single spot measurements, as shown in Figure S3 in the Supporting Information. The magnification of objective lens with numerical aperture of 0.90 was 100×, the exposure time was 1.0 s, the accumulation was 10 times, and the distance between the measurement points was 260 nm. The line scan plots were evaluated with the area intensities of their Raman bands from 2050 to 2350 cm^–1^ for CD_2_ vibration and from 2830 to 3340 cm^–1^ for CH_2_ vibration. The baselines were set based on the intensity of the ranges of 500 cm^–1^-800 cm^–1^, 1800 cm^–1^-2010 cm^–1^, and 2550 cm^–1^-2700 cm^–1^. The differential intensities of the area intensities were calculated from eq 6.
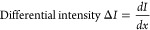
6

For the calculation of the interphase thickness, we performed more than five measurements, and the obtained values were averaged.

## Results and Discussion

**Figure 2:**
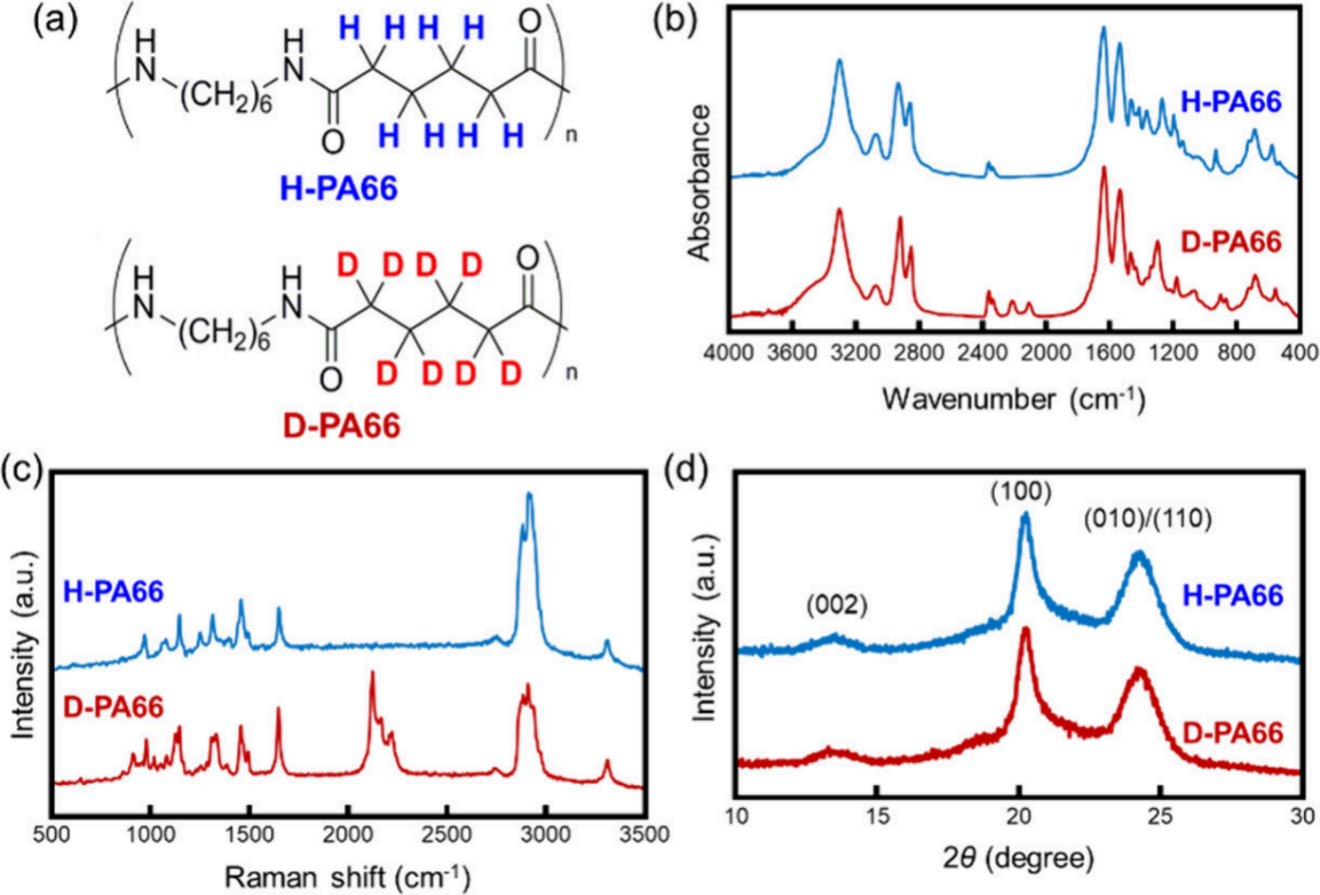
(a) Chemical structure, (b) FT-IR, (c) Raman, and (d) X-ray diffraction profiles of H-PA66 and D-PA66.

H-PA66 and D-PA66, as shown in Figure 2a, were synthesized through interfacial polycondensation in order to prepare the laminate interphase between PA66 films with identical thermal and surface properties. The synthesized polyamide 66 was characterized with ^1^H NMR measurements and FT-IR and Raman spectroscopies. In Figure S4 in the Supporting Information and Figures 2b and 2c, their ^1^H NMR profiles and FT-IR and Raman spectra are shown, respectively. In the ^1^H NMR spectrum of H-PA66, five peaks corresponding to CH_2_ groups in main chains were observed, whereas D-PA66 exhibited only three peaks due to the presence of CD_2_ groups in the adipate units. Their peaks were assigned, as shown in Figure S4 in the Supporting Information. In their FT-IR spectra, the vibration band of carbonyl groups, CH_2_ groups, and N–H of amide groups was observed at 1640, 2854, 2922, and 3306 cm^–1^.^87,88^ In the FT-IR spectrum of D-PA66, the absorption bands of CD_2_ vibration were detected at 2115 and 2212 cm^–1^.^86,87^ In addition, the Raman spectrum of D-PA66 exhibited CD_2_ vibration bands at 2121 cm^–1^, while the band was not observed in that of H-PA66 because of the absence of the CD_2_ unit in H-PA66. The intensities of CH_2_ vibration bands at 2925 cm^–1^ in the spectrum of D-PA66 were relatively lower compared with those in H-PA66. H-PA66 and D-PA66 were able to be distinguished between each other spectroscopically. In addition, the band intensities of the CD_2_ vibration in the Raman spectrum surpassed those in the FT-IR spectrum.

The differences in structural, thermal, and surface properties of H-PA66 and D-PA66 were evaluated. Figure 2d for their XRD profiles and Figure S5 and Figure S6 in the Supporting Information for their TG-DTA charts and DSC charts are shown. Tables S2 and S3 show dynamic contact angles and surface free energies. In both XRD profiles, peaks of (002), (100), and (010/110) planes of PA66 α-form were observed at 13.3°, 20.2°, and 24.1°, respectively.^89^ The crystal lattice of D-PA66 matched that of H-PA66. This means that there was no effect of the deuterated chemical structure on their crystalline structure. In the analyses of thermal decomposition behaviors of H-PA66 and D-PA66, both decomposition temperatures (*T*_d5_) were 390 °C, and their thermogravimetric trace diagrams were overlapped. The thermal decomposition behaviors of H-PA66 and D-PA66 were highly consistent regardless of the weight difference between deuterium and hydrogen atoms. In both DSC thermograms of H-PA66 and D-PA66, endothermic melting peaks were observed at 260 °C, and exothermic crystallizing peaks were observed at 228 °C. In both thermal transition behaviors of H-PA66 and D-PA66, there was also no significant difference. In addition, we investigated and compared their surface properties by evaluating surface free energies. The surface free energies of polymer substrates contribute to the interaction between polymers. Consequently, both surface free energies of H-PA66 and D-PA66 were similar to each other. Because the thermal behaviors, structure, and surface chemical affinities of deuterated PA66 are equivalent to those of nondeuterated PA66, D-PA66 is regarded as the same adherent substrate as H-PA66.

Therefore, in the analyses of the interphase of the H-PA66/D-PA66 laminates, the area intensities of their Raman bands of CD_2_ and CH_2_ vibrations at approximately 2121 and 2925 cm^–1^ were employed, and their area intensities were plotted with 1D line- or 2D map-scanning of a YAG laser. The lamination between H-PA66 and D-PA66 was performed at 250 °C for 30 s. We also have investigated the effect on lamination temperature and time. Unfortunately, the laminates prepared at a lower temperature were readily peeled during trimming with a microtome. In contrast, those prepared at a higher temperature or with a longer lamination time deformed because of complete melting, and photoluminescence was observed by the excitation of the Raman laser. Therefore, in this study, the laminating conditions at 250 °C for 30 s were employed.

**Figure 3:**
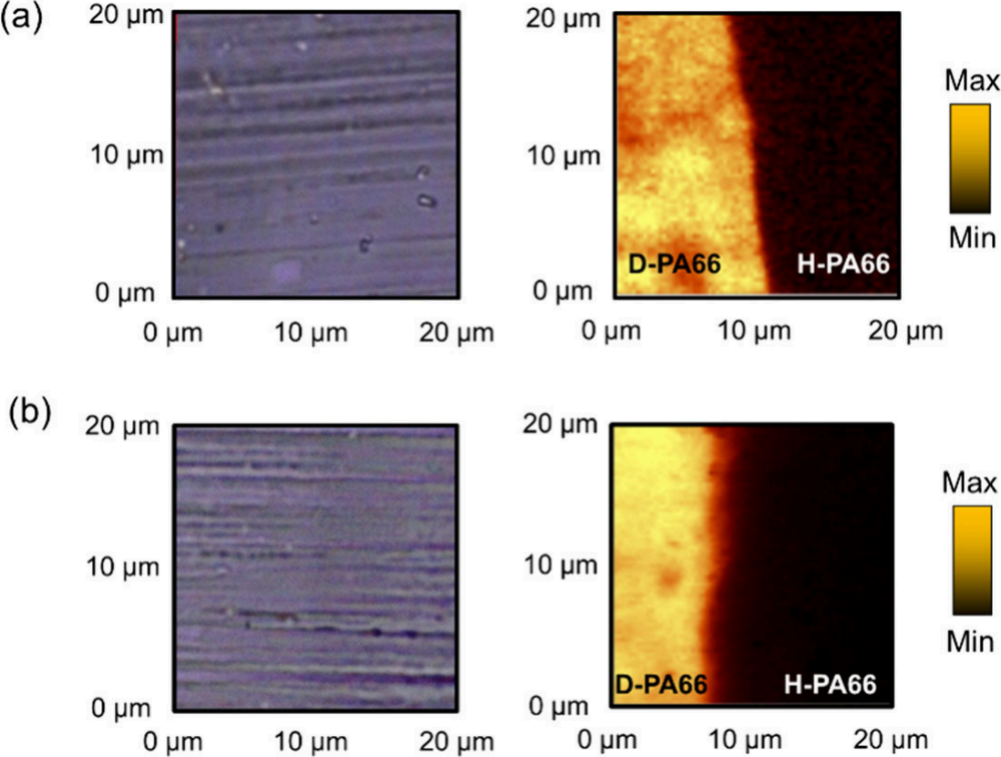
Optical microscopic images (left side) and 2D Raman mapping images of the CD_2_ stretching band (right side) of (a) s/q-Lm and (b) s/a-Lm.

We performed 2D mapping measurements of the cross section of the noncrystallized H-PA66/D-PA66 laminates both before and after annealing, denoted as s/q-Lm and s/a-Lm, respectively. Optical microscopic images and 2D mapping images of Raman intensities of CD_2_ vibration bands near the interphase at the same position are shown in Figure 3. In their optical microscopic images, there was no distinct interphase between H-PA66 and D-PA66 apparently. This reason was that it was difficult to distinguish between two materials. In contrast, the interphase of H-PA66 and D-PA66 was clearly and readily distinguished in the Raman mapping images. Through the annealing process, the contrast around their interphase was decreased, and the interphase was blurred.

**Figure 4:**
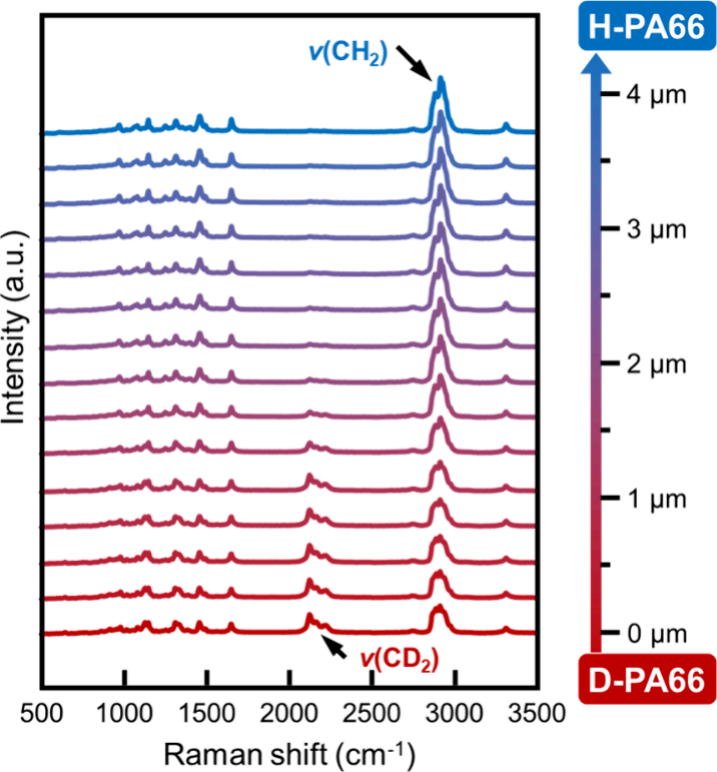
Raman scattering spectra in line scanning measurements across the s/q-Lm interphase.

**Figure 5:**
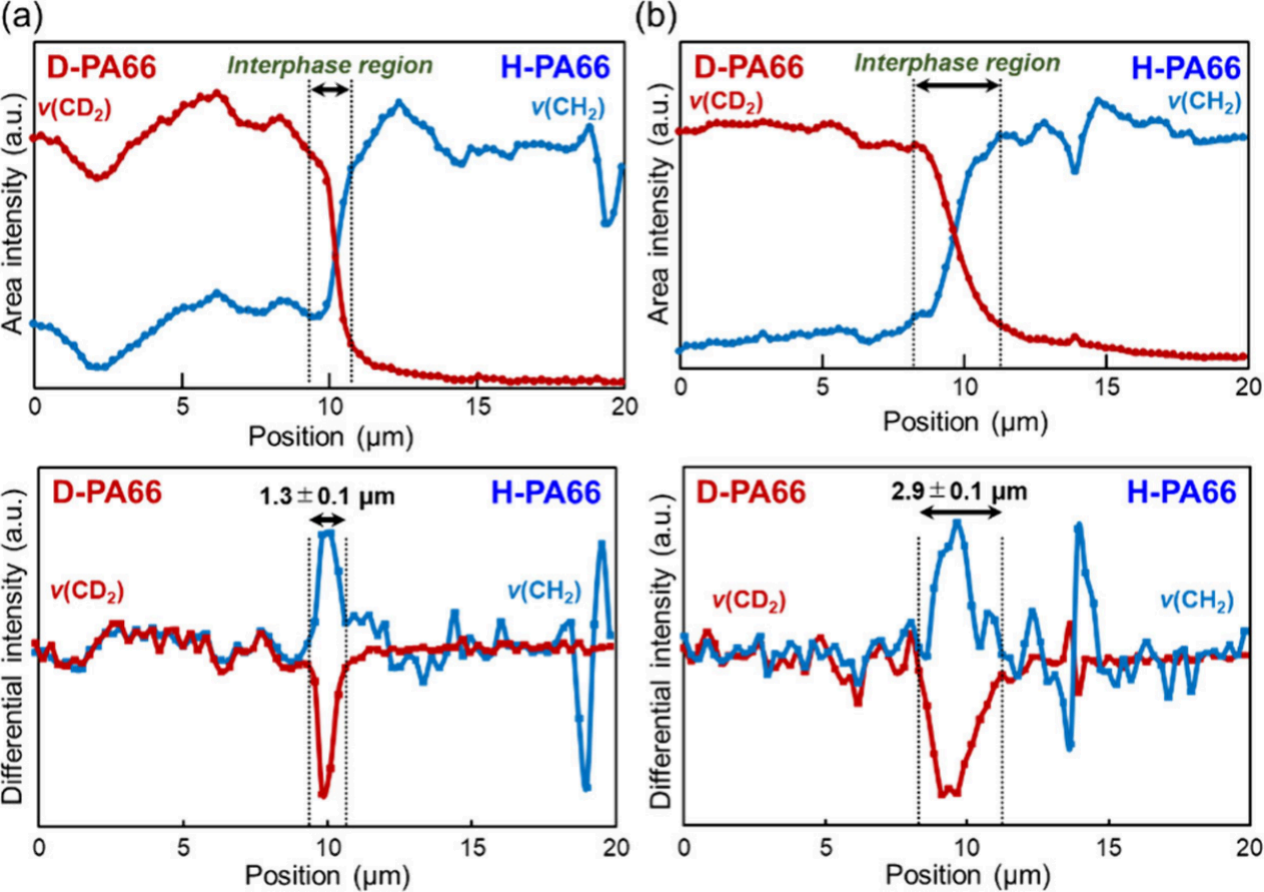
Raman scattering area intensities (upper) and differential intensities (bottom) of CD_2_ (red) and CH_2_ (blue) stretching bands at every measurement position of (a) s/q-Lm and (b) s/a-Lm.

For quantitative analyses of the distribution of the components around the interphase, line-scanning Raman spectroscopic measurements were performed along lines across the interphase of the laminated samples. The interval spacing between the measurement spots was 260 nm, similar to the spatial resolution of the Raman spectrometer. The Raman spectra at all the positions of s/q-Lm laminates in the line scanning measurements across the interphase are shown in Figure 4. In their spectra, the bands of CD_2_ vibration vanished, while the intensity of the CH_2_ vibration was increased as the measurement position transitioned from the side of the D-PA66 substrate to that of H-PA66. This shift means that the components at the measurement position were changed from D-PA66 to H-PA66 across the interphase of the laminate. In addition, the Raman spectra around the interphase encompassed both D-PA66 and H-PA66, and the interphase was a coexisting region containing both components. In Figure 5, the area intensities and their differential intensities of the Raman bands at 2121 and 2925 cm^–1^ for s/q-Lm and s/a-Lm are plotted at each measurement position. In both area intensity plots of s/q-Lm and s/a-Lm, the intensities of Raman bands for CH_2_ and CD_2_ vibrations were inverted to each other at the interphase regions. Compared with their plots at the interphase regions before and after thermal annealing, the intensities of s/q-Lm were abruptly changed in the interphase region, while a more gradual change of intensities in s/a-Lm after annealing was observed. In their differential intensities in Figure 5, the peaks were observed, where they corresponded to the region of the inversion of the area intensities for CH_2_ and CD_2_ vibrations. We defined the region of the peaks in their differential intensity plots as interphase thickness, where the CD_2_ vibration band intensities were largely changed.^18,54,55^ In addition, fluctuation behaviors of the differential intensities of the CH_2_ vibration band corresponded well to those of the CD_2_ band at the interphase regions. The laminate before annealing, s/q-Lm, possessed 1.3 μm of interphase thickness, whereas after annealing, the interphase thickness was expanded to 2.9 μm. This suggests that thermal annealing expanded the interphase region and provided the increase of the interphase thickness.

We also investigated and compared the interphase thickness of c/q-Lm and c/a-Lm, and we evaluated their annealing effects on their thickness. Their results are shown in Table 2 and Figure S7 in the Supporting Information. The averaged interphase thicknesses of c/q-Lm and c/a-Lm were 1.9 and 1.8 μm, respectively. The interphase thickness between c/q-Lm and c/a-Lm was not increased by thermal annealing.

**Table 2:** Interphase Thickness and Adhesive Fractural Energies of the Laminated Specimens

Sample	Interphase thickness (μm)	Fracture toughness (J/m^2^)
s/q-Lm	1.3 ± 0.1	451 ± 110
s/a-Lm	2.9 ± 0.1	638 ± 196
c/q-Lm	1.9 ± 0.5	59 ± 6
c/a-Lm	1.8 ± 0.1	236 ± 95

**Figure 6:**
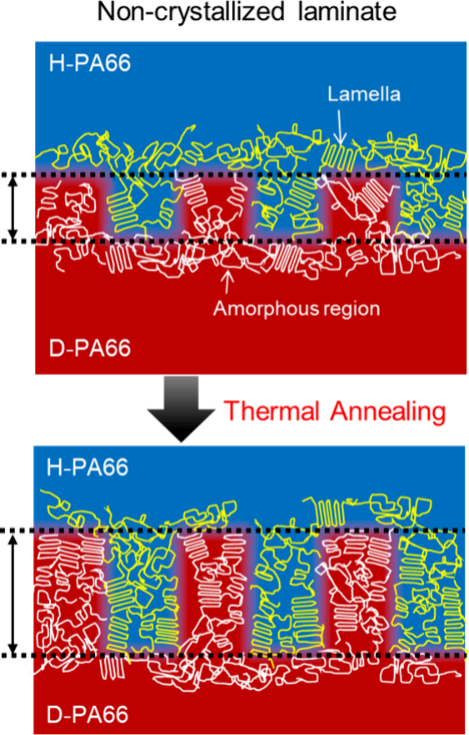
Proposal mechanism of the interphase expansion by thermal annealing.

The correlation between thermal annealing and the interphase thickness suggested that the formation of the interphase region would involve crystallization and lamella growing at the interphase, because the diffusion of polymer chains was restricted within several hundred nanometers.^54,55,90^ Therefore, laminates of the noncrystallized laminates with the highest crystallization potential expanded the interphase region after thermal annealing. We hypothesized that the formation and expansion of the interphase were attributed to both diffusion of polymer chains and crystallization and lamella growing at the interphase, as shown in Figure 6.

To investigate the correlation between adhesion properties and interphase thickness, wedge tests with razor blades were performed, and the fractural energies of the adhered specimens were evaluated. Unfortunately, their adhesion strength was not able to be accurately evaluated by single-lap shear tests and T-peel tests because fractures of the substrates were observed in their adhesion tests. The obtained fracture toughness values of PA66 laminates are summarized in Table 2. In the case of laminates of noncrystallized PA66 substrates, namely, s/q-Lm and s/a-Lm, higher fractural energies were observed than those of laminates of crystallized PA66 substrates, c/q-Lm and c/a-Lm. Moreover, thermal annealing of the noncrystallized laminated samples, s/q-Lm and s/a-Lm, enhanced the increase of fractural energy. These behaviors of adhesion fractural energies were coincident with the increase in the interphase thickness; however, the adhesion fracture toughness did not completely correspond to the interphase thickness. This discrepancy may be because the adhesion properties were obeyed by various other factors such as mechanical properties of the substrates and the interphase. Actually, the adhesion fracture energy of c/a-Lm was lower than that of s/q-Lm, but c/a-Lm exhibits a larger interphase thickness. In these cases, the adhesion properties of the noncrystallized substrates in wedge tests might be overestimated because of their low mechanical properties. In contrast, it is revealed that the adhesion properties strongly correlated with the interphase thickness in the substrates under the same conditions. The results suggest that the crystallization behavior at the single-component adhesion interphase of polyamide 66 plays a significant role in determining its adhesion properties.

## Conclusions

We performed the investigation and control of the “interphase” regions of adhesion of polyamide 66 substrates. The evaluation of their component distribution and structure at the single-component interphase is a challenge because of no difference in their chemical structure and physical properties of both adherent substrates. Herein, we evaluated the adhesion interphase between nondeuterated and deuterated polyamide 66 for investigation on the single-component adhesion interphase. Through confocal Raman scattering microspectroscopic observation, the component distribution in the interphase region was investigated. It has been proved that the interphase regions possessed a thickness of several micrometers. In addition, the interphase thickness of polyamide 66 laminates strongly depended on the thermal annealing process before and after adhesion. In the case of noncrystallized polyamide 66 substrates, the interphase thickness of their laminated specimens clearly increased by thermal annealing, whereas that with crystallized polyamide 66 substrates received no effect from the annealing process after lamination. These results supported that the mutual crystallization and the lamella interlocking at the interphase region of polyamide 66 laminates led to the formation of the adhesion interphase. The larger interphase thickness led to enhancement of adhesion properties. Even in the case of lamination with the same substrates, control of the morphology at the interphase is indispensable for the construction of the reliable adhesion system.

## Supporting Information Available

Wedge test image, NMR charts, thermogravimetric traces, DSC thermograms, crystallinity, dynamic contact angles, surface free energies, schematic image of line-scanning Raman measurements, plots of Raman scattering intensities and differential intensities, and X-ray diffraction profiles (PDF)
