# Supplementary material for: Observation and Control of Single-Component Adhesion Interphase of Polyamide 66 through Confocal Raman Microspectroscopy
Source: ACS Appl Mater Interfaces. 2024 Dec 26;17(13):20374–82. doi: 10.1021/acsami.4c18513 (PMC11969429; doi:10.1021/acsami.4c18513)
Supplement: Supplementary file 1 — am4c18513_si_001.pdf [file am4c18513_si_001.pdf]

Supporting Information

Observation and Control of Single-Component Adhesion Interphase of Polyamide 66 through Confocal Raman Microspectroscopy

Takuya Matsumoto, Naoki Shimoura, Naho Aoki, Naoto Takahashi, Shun Mizuno, and Takashi Nishino**

Department of Chemical Science and Engineering, Graduate School of Engineering, Kobe University, Rokko, Nada, Kobe 657-8501, Japan

*(T.M.) E-mail: matsumoto0521@person.kobe-u.ac.jp

*(T.N.) E-mail: tnishino@kobe-u.ac.jp

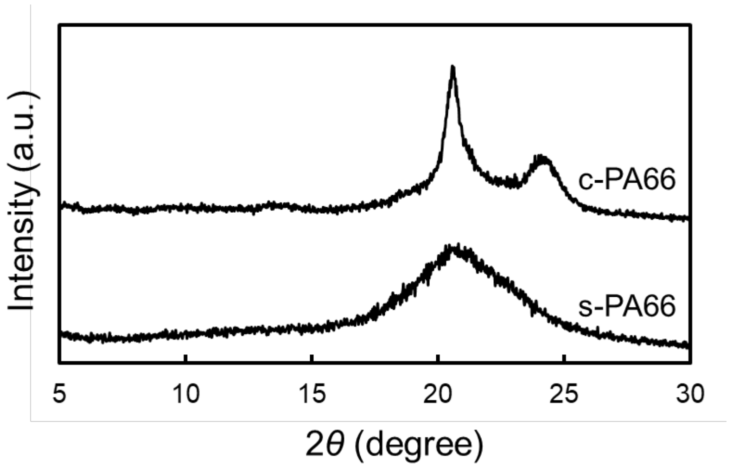

Figure S1. X-ray diffraction profiles of s-PA66 and c-PA66 substrates.

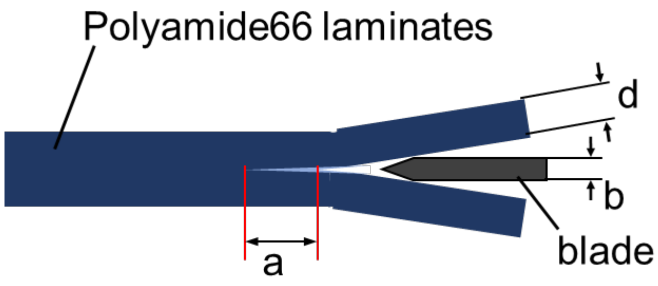

Figure S2. Specimen model in wedge test.

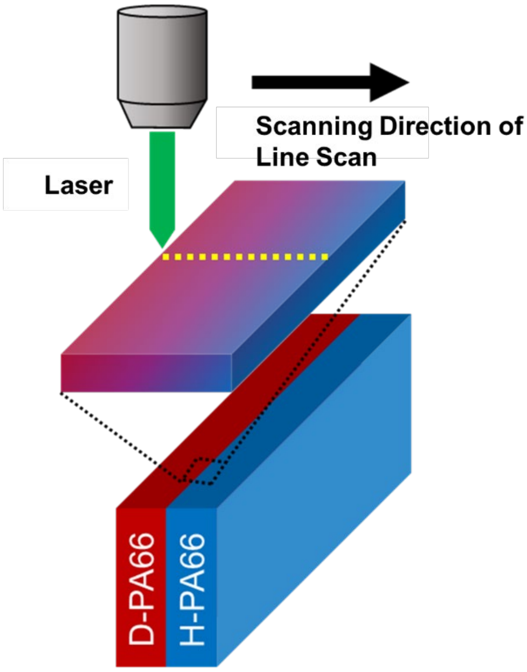

Figure S3. Raman scattering measurements of laminated PA66 specimens.

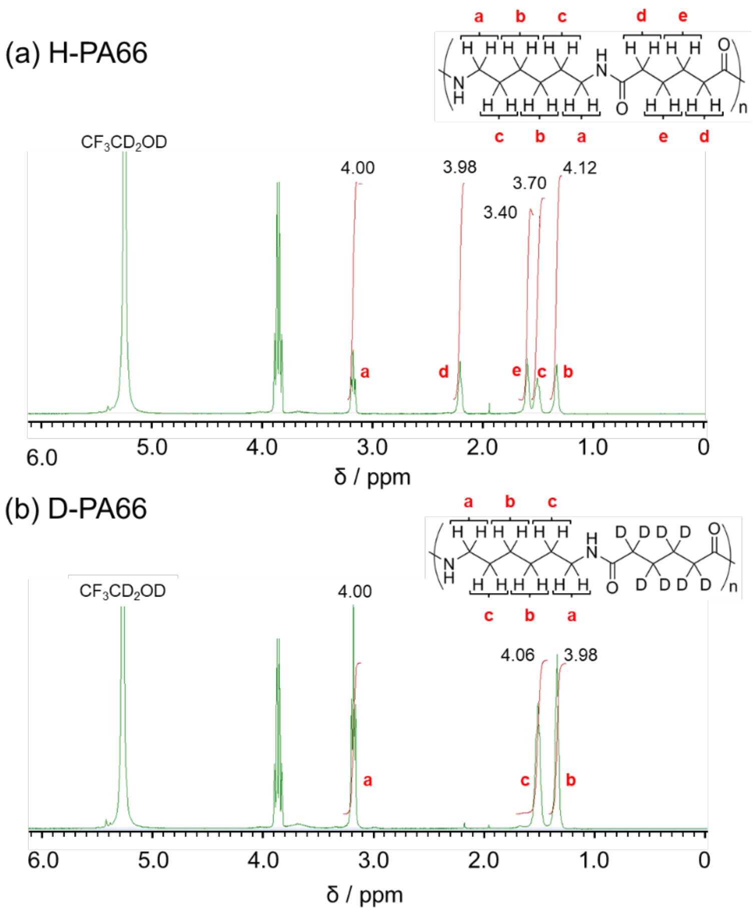

Figure S4. ^{1}H-NMR charts of (a) H-PA66 and (b) D-PA66 in 2,2,2-trifluoroethanol-*d3*.

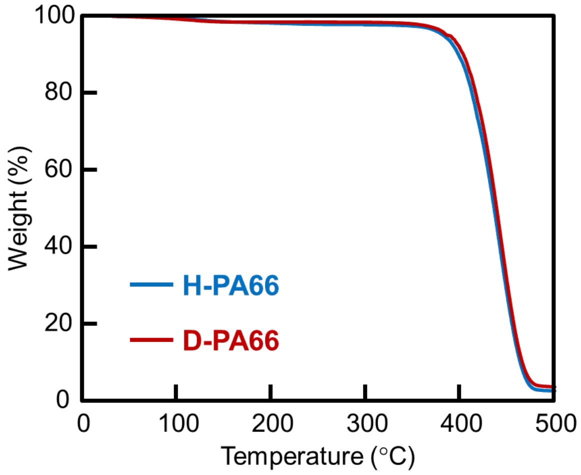

Figure S5. Thermogravimetric traces of H-PA66 and D-PA66.

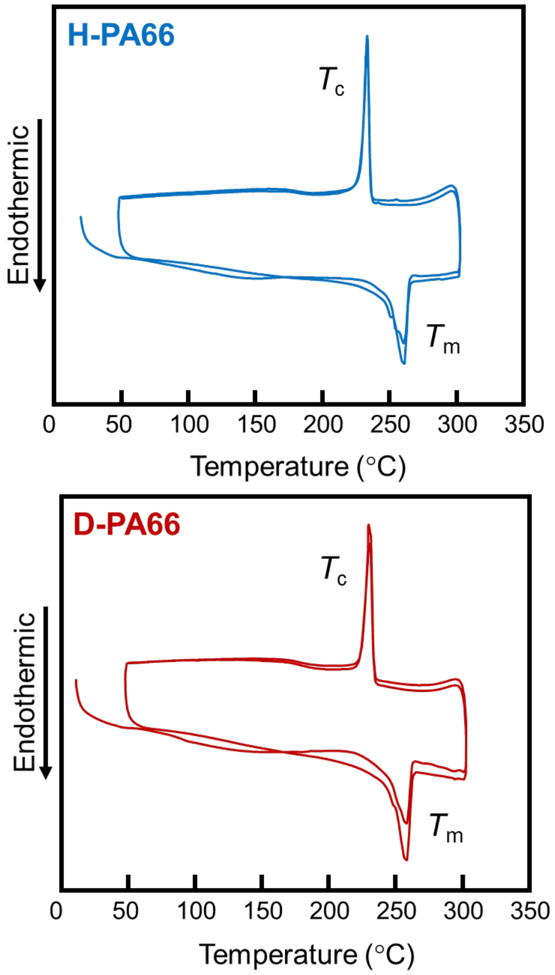

Figure S64. DSC thermograms of H-PA66 and D-PA66

Table S1. Crystallinity of H-PA66 and D-PA66 evaluated by X-ray diffraction and DSC.

	X_c (XRD)	X_c (DSC)	
		1st cycle	2nd cycle
	%	%	%
H-PA66	36.5	37.8	48.5
D-PA66	35.7	35.1	45.7

Table S2. Dynamic contact angles of droplets of water and diiodomethane of H-PA66 and D-PA66.

	water	CH_2I_2
	Degree (θ_a / θ_r / θ_{av})	
H-PA66	71.6 / 14.5 / 50.0	33.7 / 12.6 / 25.3
D-PA66	71.9 / 13.7 / 50.1	38.5 / 16.6 / 29.5

Table S3. Surface free energies of H-PA66 and D-PA66.

	γ	γ^{d}	γ^{p}
		mJ/m^2	
H-PA66	55.6	35.7	20.0
D-PA66	54.7	34.0	20.7

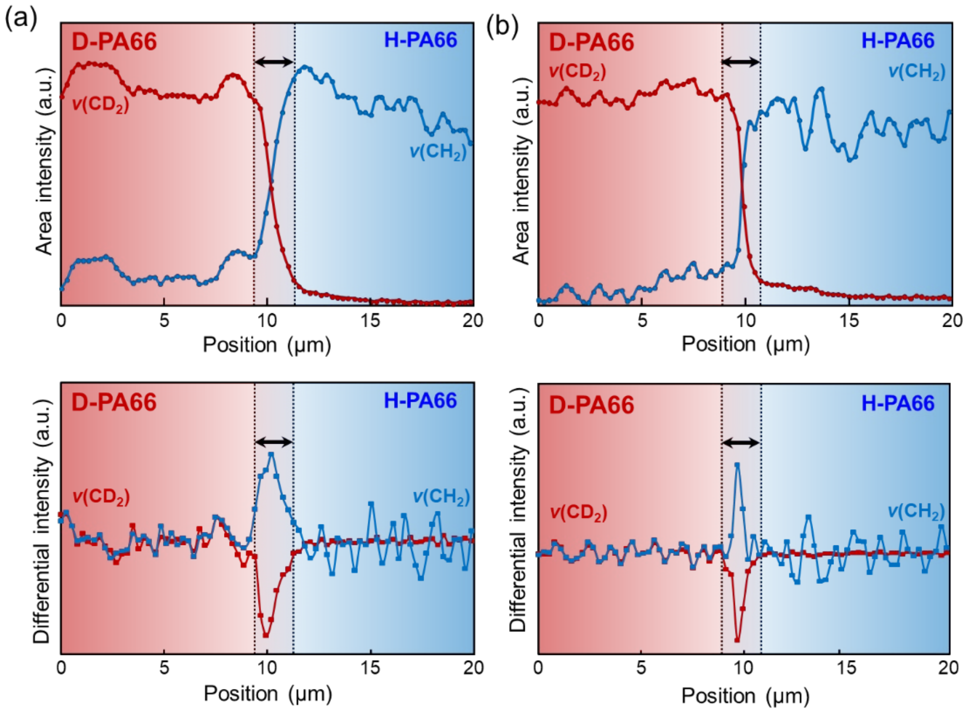

Figure S7. Raman scattering intensities (upper) and differential intensities (bottom) of CD_2 stretching band at every measurement position of (a) c/q-Lm and (b) c/a-Lm.
